# To the Editors of the London Medical and Physical Journal

**Published:** 1818-10

**Authors:** J. Austin

**Affiliations:** North Chapel, Sussex


*To the Editors of the London Medical and Physical Journal.*

GENTLEMEN,

I SHOULD not have thought of giving publicity to the following case, but for a remark I met with in a work, more than probable in the hands of many young practitioners:—

"Many might suppose bark to be a specific for the imperfect periodical amaurosis; this, however, is not the case. Bark, which is efficacious in intermittent fevers, and other periodical diseases, far from *curing* the periodical amaurosis, *seems to exasperate it*, rendering its return more frequent and of longer duration than before."—Vide *Cooper's Surgical Dictionary*, p. 16, edit. 3.

If you think the case annexed, contradicting so materially the above assertion, worthy the notice of the profession, and will insert it in your valuable publication, you will oblige,

Gentlemen,

Your obedient servant,

*North Chapel, Sussex;* J. AUSTIN.

*July* 8, 1818.

Sarah Madgwick, aged twenty-four, of delicate habit, mother of two children, with each of whom she had easy labours, about the close of last year first complained of a little dimness of sight, and noticed she was usually affected with it in the afternoons and evenings. She paid but little attention to it for some time, till she observed that about noon every day she began to experience acute sensations of pain, darting, as she said, across both eyes, that these pains continued some time, and were invariably followed, first by dimness, and at length with such a loss of sight, that she could just distinguish a candle in the room, or the light of the fire, but no other objects: she would go to bed in this state, but rise in the morning perfectly free from complaint, save a slight sense of stiffness or weight, as she expressed it, of the upper eyelids. Her sight, during the morning, was strong and good, and without any appearance, as far as I could learn from herself or friends, denoting the least affection of the eyes.

Things went on thus for two months: being in poor circumstances, she had not applied for any advice, and, her health being good, she entertained hopes of getting better. The constant and regular daily return of these attacks, however, thwarted her expectations, and she at length consulted an eminent surgeon in this neighbourhood, under whose care she continued some time without the least benefit. It is about two months since I first saw her, in the morning, about two hours before her usual daily attack; she was then, as ever since, in good bodily health; there was no appearance whatever of any affection of either eye, if we except the pupils being rather more contracted than natural, but the sensibility of the iris was unimpaired. At first I ordered a mixture of equal parts aq. ammon. pur. and lin. camph. comp. to be applied three or four times a-day on the temples and over the upper eyelids, and three 5-grain pills of the pilul. ferri cū myrrha to be taken twice a-day. In about a week after, I directed two blisters to be applied to the temples. These remedies, however, afforded not the least relief, and, as the case appeared to me to be an intermitting amaurosis, I determined to lose no more time, but try the effects of the cinchona; I thought it prudent, however, previous to its exhibition, to clear the stomach and *primæ viæ*, by a solution of antim. tart., which I ordered to be taken early in the morning, so as to produce, first, full vomiting, and afterwards in nauseating doses, till the expected attack at noon: she complied with my wishes, continuing in a state of nausea from six in the morning till noon, when the pains, &c. returned as usual, and no more was taken: I then ordered ʒi. pulv. cinchon. to be taken every day at eleven o'clock; she continued these four days without any alteration. Never having seen her when under the influence of these attacks, as she lived some distance from me across the country, out of my line of practice, I determined to ride over to her, with a view of remarking any alteration in the appearance of the eyes the attacks might induce: I arrived at her residence about two o'clock; it was the fifth day from commencing the bark, and I was agreeably surprised to find that, for the first time for nearly five months, she had not then had the attack, neither had she felt any of the usual preceding pains. I waited with her as long as I could spare the time, nearly half an hour, without any of her usual symptoms appearing; I desired her by all means to continue her powders, and manage them so as to take one, as near as she could, about an hour before the expected attack.

The latter end of the week she came to me to say, that, on the day I saw her, the attack did not come on till after three o'clock, that it was not preceded by the usual pains, and that, during the whole of that afternoon, she was not blind as usual, but experienced only an unpleasant dimness. The next day, she took her dose at two o'clock; the attack did not come on till five. The seventh day she intended to have taken it at four o'clock, but the attack, still slight, came on at two o'clock. The day following, she took it at twelve o'clock, and was totally free from any symptom till between six and seven the evening, when a little dimness came on, but not so as to prevent her continuing to attend to her household affairs. To conclude, she continued the bark regularly every day afterwards, for about a fortnight from commencing; when, being two days without any attack whatever, she took it only on alternate days, about another week, and at length totally discontinued it. She has not taken any thing now above a month; has continued free from every complaint; and I observe each pupil, since she has been relieved, appears more dilated than when I first saw her.

May I enquire whether the early efficacy of the cinchona may not, in some measure, be attributed to the previous action of the antim. tart ?*

---

*For the London Medical and Physical Journal.*

### *On Specific Gravity*; by Nicholas Littleton, Esq.

* We consider the author is correct in this conjecture: the accumulation of irritability which took place during the inaction accompanying the state of nausea, doubtless, contributed much to increase the effects of the bark. We have seen cases of intermittent fever that have resisted the use of cinchona, exhibited in the usual way, which have quickly yielded to it, when given after a state of nausea had been kept up for several hours.—Edit.

